# Management of oxygen saturation monitoring in preterm newborns in the NICU: the Italian picture

**DOI:** 10.1186/s13052-021-01050-3

**Published:** 2021-05-03

**Authors:** Serafina Perrone, Maurizio Giordano, Giuseppe De Bernardo, Paola Lugani, Pasquale Sarnacchiaro, Gemma Stazzoni, Giuseppe Buonocore, Susanna Esposito, Maria Luisa Tataranno, on behalf of the National Study Group of Neonatal Clinical Biochemistry of the Italian Society of Neonatology, Simona Pesce, Lidia Grappone, Marina Riccitelli, Sara Cornacchione, Nicola De Virgilio, Elisa Laschi

**Affiliations:** 1 Department of Medicine and Surgery, University of Parma, Parma, Italy; 2 Department of Clinical Medicine and Surgery, University of Naples Federico II, Naples, Italy; 3 Division of Pediatrics Neonatology and NICU, Ospedale Buon Consiglio Fatebenefratelli, Naples, Italy; 4 Department of Legal and Economic Sciences, University of Rome Unitelma Sapienza, Rome, Italy; 5 Department of Molecular and Developmental Medicine, University of Siena, Siena, Italy; 6 Department of Neonatology, Utrecht University Medical Center, Utrecht, The Netherlands

**Keywords:** Neonate, NICU, Oxygen saturation

## Abstract

**Background:**

Although many studies emphasize the importance of using oxygen saturation (SpO_2_) targets in the NICUs, there is a wide variability in used saturation ranges among centers. Primary aim was to draw a representative picture on how the management of oxygen monitoring is performed in the Italian NICUs. Second aim was to identify healthcare-professionals related factors associated with oxygen targeting in the preterm population.

**Methods:**

Cross-sectional study with data collection via an electronic survey form. A questionnaire containing pre-piloted and open questions on monitoring and management of the SpO_2_ was administered to neonatologists across the network of the Italian Society of Neonatology. The questions focused on: the infrastructure, specific training, healthcare professionals and patients-related factors. The results of the survey were anonymously collected, summarized and analyzed.

**Results:**

Out of 378 questionnaires, 93 were correctly filled. Thirty-six different SpO_2_ ranges were observed. Centers using written standard operating procedures on oxygen management and SpO_2_ monitoring maintained a correct average range of SpO_2_ 90–95%, avoided hyperoxia and reconsidered saturation targets in relation to comorbidities. 39.8% of responders disabled alarms during neonatal care. One center used biomarkers for complete monitoring of neonatal oxygenation status.

**Conclusions:**

There is considerable variation in SpO_2_ targets for preterm infants in the Italian NICUs. Standard operating procedures and specific training for health care personnel are the main factors playing a role for the correct maintenance of the recommended oxygen targets in preterms.

## Background

Oxygen is essential for aerobic life, but it can be considered a double-edged sword in the perinatal period having both positive biological benefits and toxic effects. Oxygen toxicity is due to the development of reactive oxygen species (ROS), potent oxidants in biological fluids that may damage tissues, through reaction with lipids, proteins, DNA, amino acids and several other molecules [1]. An imbalance between oxidants and antioxidants is called oxidative stress: a potential cause of cell damage [2]. Newborns, especially if preterm, are particularly susceptible to oxidative stress due to the immature antioxidant capacity and the likely exposure to many processes such as hypoxia, hypoxia-ischemia, hyperoxia and infections, leading to high levels of free radicals’ production [3]. Hypoxia and hyperoxia predispose preterm newborns to oxidative stress, through the free radical generation. Antioxidant capacity is lower in the newborn and particularly in the premature infant in comparison to term newborn [2, 3]. Therefore, premature infants are especially prone to oxidant injury, in various organs and systems such as lungs, retina and erythrocytes [4–6] with short and long term effects. The careful monitoring of oxygen saturation (SpO_2_) levels during neonatal intensive care unit (NICU) admission is of outmost importance, in order to avoid excessive and undesired exposure to hypoxia/hyperoxia [7, 8]. Clinical studies underlined how the level of generally accepted saturation targets for children and adults could not be considered acceptable for preterm and low birth weight infants [9, 10]. Recently, multicenter randomized controlled trials support the recommendation to keep SpO_2_ between 90 and 95%, in infants with a gestational age less than 28 weeks, up to 36 weeks post-menstrual age [11–13]. However, wide variability in SpO_2_ ranges has been reported and there is no consensus yet on the specific prerequisites for the management of SpO_2_ monitoring in the NICUs [14, 15]. There is current evidence on the presence of the condition of “alarm fatigue” which is the desensitization of health care practitioners to the thousands of alarms of a single day in the NICU [16]. Fatigue is related to the many false alarms generated by pulse oximeters; each alarm is supposed to be associated with active nursing intervention with a frequency of around 5–10 min [17, 18]. Nurses have to face this dilemma, and previous papers already showed that they tend to disregard alarm policy, due to the high number of false alarms [19]. More attention is needed in selecting reasonable alarms’ ranges and levels, while the risk of missing or delaying response to important events must be also considered [20]. Moreover, there is an increasing tendency to disregard the high alarm limit, with the assumption that hypoxemia is more detrimental than hyperoxemia. A recent survey on pulse oximeter saturation target limits for preterm infants in European NICUs pointed out the present climate of uncertainty regarding the optimum range of pulse oximeter SpO_2_ for preterm infants, reporting wide institutional variations on SpO_2_ targets [21]. The aim of this study is to characterize how SpO_2_ is monitored in preterm infants in the Italian NICUs. A further aim is to identify healthcare-professionals related factors associated with different oxygen targeting in preterm newborns.

## Materials and methods

This is a national, multi-center, cross-sectional study, implemented by the scientific working group of “Clinical neonatal Biochemistry” of the Italian Society of Neonatology.

Data collection was performed using an electronic survey (eCRF) sent to all the chairs of italian NICUs. Contacts were obtained from the registry of the members of the Italian Society of Neonatology. The questionnaire was also sent to all the neonatologists working in these units.

Data collection was performed using Survey Monkey software (SurveyMonkey Inc. San Mateo, California, USA). The first survey was sent in December 2017. A reminder with a new link was sent in February 2018. Questionnaires were administered and collected anonymously. In order to check reliability and consistency of the answers, redundant questions were included in the survey (i.e. question number 18, q 2 in training related variables, q 9 in staff variables etc.). Exclusion criteria were: an incomplete survey for more than 50%, inconsistent answers to redundant questions more than two times in the whole survey.

The eCRF consisted of two parts (Table 1). Part one contained general information about the hospital, the NICU, the patient–nurse ratio and implemented treatment strategies (biomarkers measurement, standard operating procedures, guidelines). Part two enquired specific information about the management of SpO_2_ monitoring currently used in the unit. The following variables were also assessed through the eCRF: infrastructure variables, training, technology variables, staff variables and patient-related variables. For each section multiple choice as well as open questions were included. The respondent neonatologists were instructed, via an introductory email, to provide answers reflecting their unit practice, based on local protocols/standard care, and not personal preferences. A reminder questionnaire was sent once to the nonresponding neonatologists.

**Table 1:** Electronic survey sent to the Italian neonatologists

Infrastructure variables	
1. **Your NICU is based in a:**	2. **Your NICU is:**
▪ II level hospital	▪ South of Italy
▪ III level hospital	▪ North of Italy Center of Italy
3. **Is yours a University hospital?**	4. **Indicate the annual number of births in your hospital**
▪ Yes	
▪ No	
5. **Indicate the annual number of newborns with gestational age < 32 weeks assisted in your NIC**	
6. **Indicate the annual number of newborns with gestational age < 28 weeks assisted in your NICU**	
7. **How many NICU beds does your NICU have**	8. **Which are the doctors/beds ratio in your NICU?**
9. **Which is the nurses/beds ratio in your NICU?**	10. **Is there a local oxygen management protocol in your NICU?** ▪ Yes ▪ No ☐
11. **Indicate the minimum and maximum values of the range of SatO2 used in its NICU for newborns with gestational age less than 32 weeks requiring oxygen supply**	
12. **In which conditions may the above ranges vary?**	
▪ Never	
▪ Variation in ventilator support mode (e.g. from not invasive to invasive)	
▪ Presence of associated comorbidity (anemia, congenital cardiopathy, retinopathy of prematurity, need for surgery, sepsis)	
▪ Other (explain here your answer)	
13. **In case of SpO**_**2**_ **range variation, which of the two alarms is modified?**	
▪ Lower value alarm	
▪ Upper value alarm	
▪ Both	
14. **Who is in charge of setting the minimum and maximum alarms?**	
▪ Chief	
▪ Neonatologist	
▪ Nurse	
15. **Who is in charge to change the alarm value?** (You can choose more than one answer)	16. **Who is in charge to disable the maximum alarm?** **(You can choose more than one answer)**
▪ Chief	▪ Chief
▪ Neonatologist	▪ Neonatologist
▪ Nurse	Nurse
17. **In which conditions are the alarms disabled?** **(You can choose more than one answer)**	
▪ Never	
▪ During invasive procedures (such as CVC insertion, chest drainage, reintubation)	
▪ During nursing care (washing, weight evaluation, change of the diaper)	
▪ patient respiratory instability	
▪ Other (explain here your answer)	
18. **If the alarms are disabled, which of the two alarms is disabled?**	
19. Lower alarm	
20. Upper alarm	
21. Both	
22. **Who responds to the alarm signal?**	23. **Is there written documentation of the interventions in**
▪ Doctor on duty	24. **response to the alarm signal?**
▪ Nurse	▪ Yes
▪ Indifferently the doctor or the nurse	▪ No
**Training related variables**	
1. **Is there a staff training program on the use of the pulse oximeter and on the rationale for careful monitoring of O2 saturation?**	
▪ Yes	
▪ No	
2. **Is there a formal staff training on how to respond to alarms?**	
▪ Yes	
▪ No	
**Technology variables**	
1. **Indicate the type of the pulse oximeter in use in your NICU**	
2. **Is an O**_**2**_ **saturation daily plot available ​​for admitted newborns?**	
▪ Yes	
▪ No	
3. **If you answered yes to the previous question, is it possible to archive daily data?**	
▪ Yes	
▪ No	
**Staff variables**	
1. **Do you think that a high alarm frequency during the work shift leads to latency in response time to the alarm or a decreased attention to that?**	
▪ Yes	
▪ No	
2. **If you answered yes, for what kind of alarm do you think that happens**?	
3. Lower alarm	
4. Upper alarm	
5. Both	
6. **In case of severe conditions, with frequent activation of the alarm, is there a progressive latency in the response time to the upper value alarm?**	
▪ Yes	
▪ No	
7. **Is the acoustic intensity of the alarms reduced during the night shift?**	8. **During the night shift, can the upper value alarm be changed / disabled?**
▪ Yes	▪ Yes, it can be changed
▪ No	▪ Yes, it can be disabled
	▪ No
9. **1Are alarms disabled during assistance maneuvers (e.g. washing, suction, weight evaluation, nursing care)?** ▪ Yes	
▪ No	
10. **If you answered yes, how long are they disabled on average?**	11. **During the execution of the assistance maneuvers, does the healthcare professional use supplemental oxygen?**
▪ Less than 5 min	▪ Yes
▪ From 5 to 10 min	▪ No
▪ More than 10 min	▪
12. **Before carrying out invasive maneuvers (e.g. reintubation, positioning of the thoracic drainage, CVC insertion, etc.) is a further supplementation of oxygen used, compared to that already administered?** ▪ Yes ▪ No	
**Patient-related variables**	
1. **How many newborns less than 32 weeks of gestational age with respiratory support are currently hospitalized in your NICU?**	
2. **How many of the newborns mentioned in the previous question are assisted with non-invasive ventilation?**	3. **How many of the newborns mentioned in the previous question are assisted with invasive ventilation?**
4. **Indicate the minimum and maximum values ​​of the SatO2 range used for currently hospitalized newborns with GA < 32 weeks**	
5. **Do the clinical conditions of the newborn (anemia, hypotension, apnea, infections, need for mechanical ventilation) influence the saturation range set?**	6. **In your NICU, do you have NIRS as an additional tissue oxygenation monitoring system?** ▪
▪ Yes	▪ Yes
▪ No	▪ No
7. **In your NICU, do you have the VEGF dosage as an additional tissue oxygenation monitoring tool?**	8. **Is there the possibility to measure oxidative stress by dosing specific biomarkers?**
▪ Yes	▪ Yes
▪ No	▪ No

*NICU* neonatal intensive care unit; *SpO2* oxygen saturation; *CVC* catheter venous central; *VEGF* Vascular-Endothelial Growth Factor; *NIRS* near infrared spectroscopy

### Statistical analysis

Data analysis was performed using IBM SPSS Statistics (IBM Corp., Armonk, NY, USA). Normal distribution of data was evaluated by Kolmogorov Smirnov test. The two-proportions z-test was used to compare two observed proportions and the two-sample t-test to test whether the means from the two populations were equal or not. All tests were conducted two-sided in an explorative manner on a 5% significance level. Logistic regression model was performed to identify factors independently associated with the use of oxygen saturation monitoring modalities. Based on these statistical models, odds ratio estimates (OR) were calculated with 95% confidence intervals (CI).

## Results

**Fig. 1:**
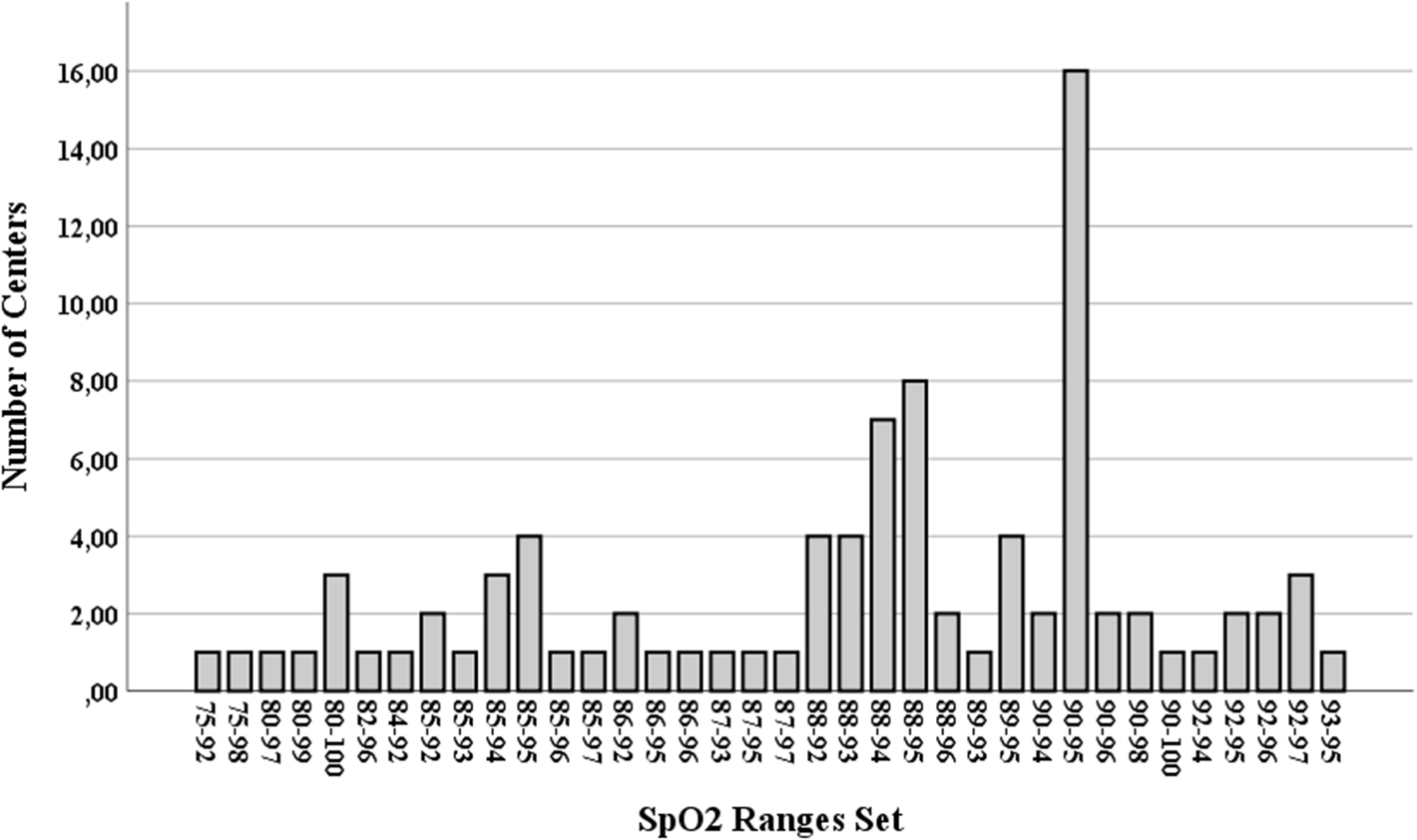
SpO_2_ ranges used in participating NICUs

**Fig. 2:**
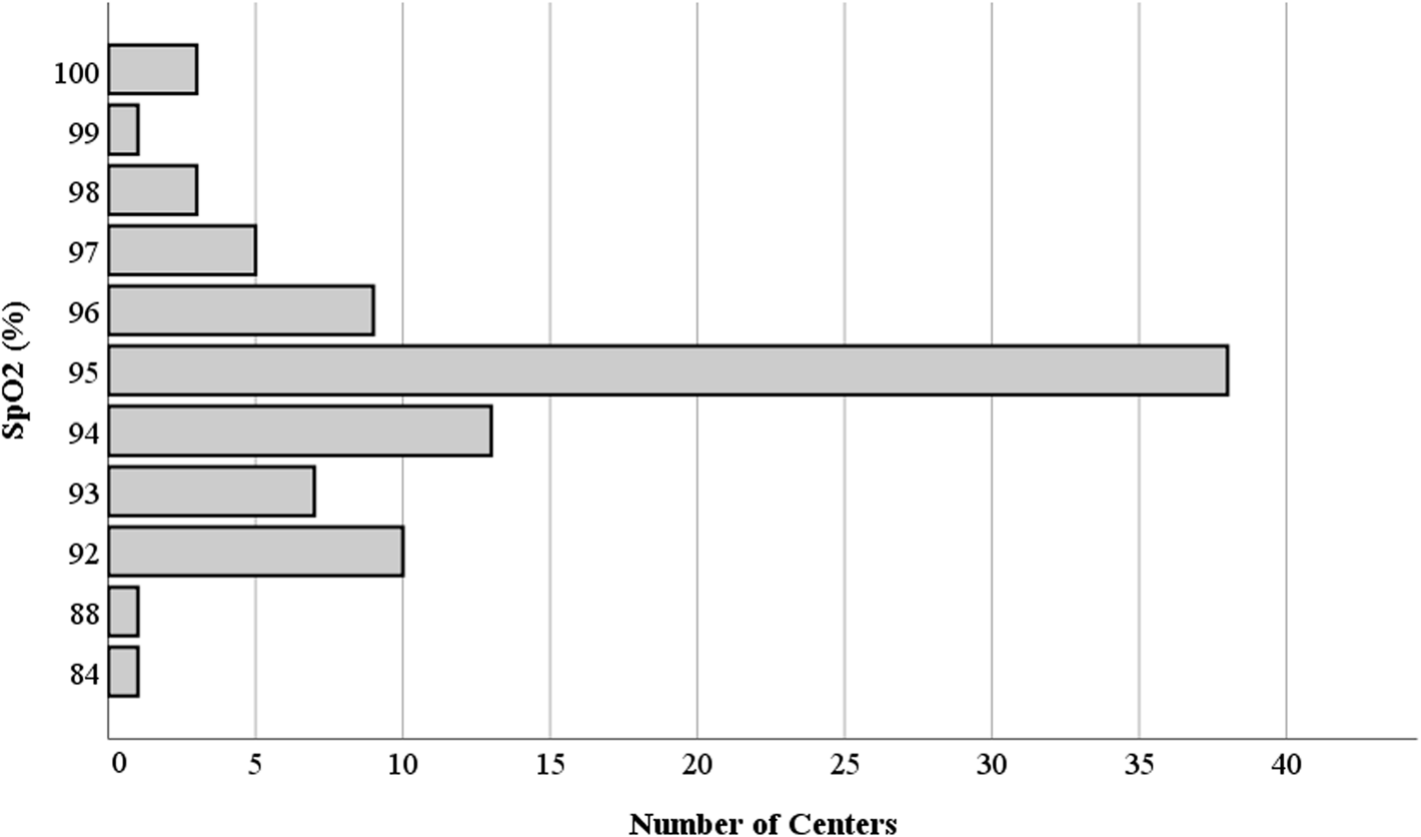
Maximum SpO_2_ desired level used in participating NICUs

**Fig. 3:**
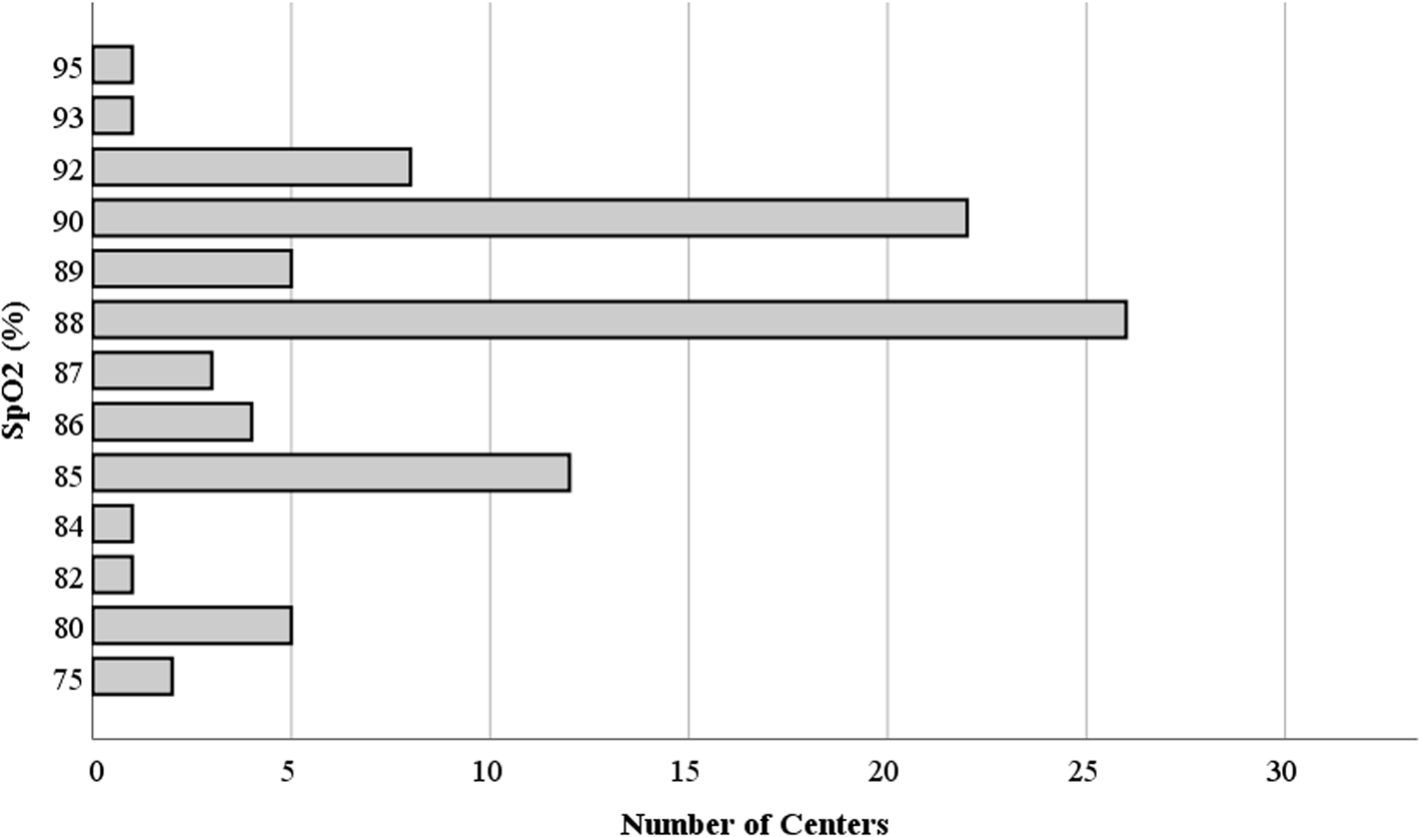
Minimum SpO_2_ desired level used in participating NICUs

**Fig. 4:**
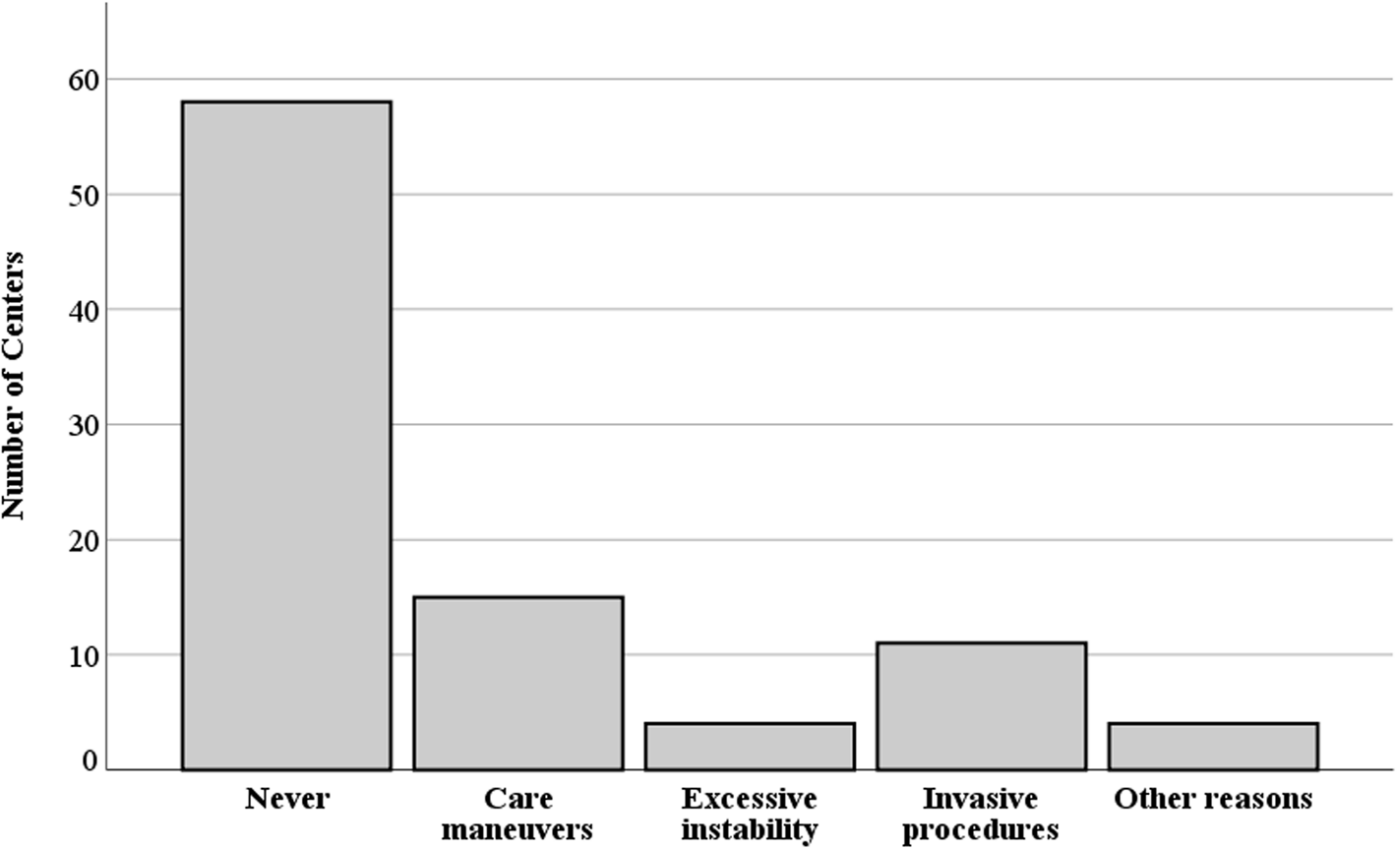
Situations in which alarms are disabled

Out of a total of 378 questionnaires sent, 104 replies were received. Of these only 93 were complete and showed consistent answers and therefore assessable for our study (24.6% of the questionnaires sent). The characteristics of participant centers are described in Table 2. Over the total 93 different NICUs, 36 different SPO_2_ ranges were reported, with wide variability between the minimum and maximum target levels. The most frequently used range of SpO_2_ was 90–95% (16 centers), 88–95% (8 centers) and 88–94% (seven centers) (Fig. 1). The range of maximum SpO_2_ used levels varied from 100% (four centers) to 92% (10 centers) (Fig. 2). The range of minimum SpO_2_ levels varied from 75% (two centers) to 93% (one center) (Fig. 3). In 64.4% of the centers the lower limit of SpO_2_ is set below 90%; in contrast, in 24.4% of the centers the upper limit of SpO_2_ is set above 95%. NICUs using written standard operating procedures or specific local guideline on oxygen management and SpO_2_ monitoring, maintained average maximum desired levels of SpO_2_ of 94% + 2 versus 96% + 2 of centers that did not have a written local protocol (*p* = 0,003). However, there were no statistically significant differences with regards to the mean values set for the desired minimum SpO_2_ (88% ± 2 versus 88% ± 3, respectively; p = 0,143). Similarly, centers performing specific training of health care personnel on management of oxygen monitoring, would set the desired maximum values of SpO_2_ on average at 94 compared to the average values of 95% of the centers not performing any training (*p* = 0.037). There were no statistically significant differences in the mean values set for the desired minimum SpO_2_ (minimum SpO_2_ = 88% ± 2 versus 88% ± 3, respectively; p = 0,662). SpO_2_ desired range could be changed in 56% of participating NICU in case of associated comorbidity (e.g. anemia, cardiopathy, ROP, BPD, surgery, sepsis), in 13.2% of cases also if the ventilatory support mode was changed, in 12.1% of cases in other conditions, such as in case of both comorbidity and variation of the ventilatory support mode (seven centers, 70%), in case of no oxygen supplementation (three centers, 30%). One center modified the maximum limits, by increasing it, in case of corrected gestational age > 32 weeks. In 18.7% of the centers, SpO_2_ limits were never changed. SpO_2_ alarms were never turned off in 63.4% of participating centers (*n* = 59). They were disabled during care maneuvers in 16% (*n* = 15), during invasive procedures in 11.8% (*n* = 11), excessive instability of the patient in 4.3% (*n* = 4), other reasons in 4.3% of the centers (one center in the assistance of terminal patient to death, two centers in case of no supplemental oxygen, one center in case of both invasive procedures and care maneuvers) (Fig. 4). Centers that performed staff training on the monitoring of the SpO_2_ turned off the alarms during the assistance maneuvers less frequently compared to those with the opposite attitude (23% versus 76%, respectively; OR (95% CI) =0,368 (0,001-0,269); *p* = 0.047). Twenty-six centers (five of second level and 21 of third level) had NIRS technology available to study cerebral SpO_2_. Four third level centers had the possibility to dose VEGF. Thirteen centers (three of second level and ten of third level) measured biomarkers of oxidative stress. In a single third level and university center all the three adjunctive technologies were available for the complete study of the oxygenation status of the newborn.

**Table 2:** Characteristics of participating centers

		N	Percentage
**Participants NICU**		93	100%
**NICU based in hospital in the**	South of Italy	29	31,2%
	Center of Italy	26	28%
	North of Italy	38	40,8%
**Number of births / year (mean)**		2120	–
**Total number of newborns with gestational age < 32 weeks assisted in NICU/ year (total n°)**		6628	–
**Total number of newborns with gestational age < 28 weeks assisted in NICU/ year**		2757	–
**Number of NICU beds (mean)**		10	–
**Median doctors/beds ratio in NICUs**		1:4	–
**Median nurses/beds ratio in NICU**		1:4	–
**Oxygen management protocol in NICU**		59	63.4%
**Staff training program on the use of the pulse oximeter and on the careful monitoring of O**_**2**_ **saturation**		49	52.7%
**Formal staff training on how to respond to alarms**		38	40.9%
**Who is in charge to set the minimum and maximum alarms?**	Neonatologist	78	83.9%
	Nurse	11	11.8%
	Chief	4	4,3%
**Who is in charge to disable the maximum alarm?**	Neonatologist	73	88.2%
	Nurse	13	13%
	Chief	4	4.3%
**In which conditions when the alarms can be disabled?**	Never	59	63.5%
	During the execution of invasive procedures	11	11.8%
	During the nursing care	23	24.7%
**Time-length of alarms disabled during the nursing care**	Less than 5 min	28	30.1%
	Between 5 and 10 min	7	2.2%
	More than 10 min	2	7.5%

*NICU* neonatal intensive care unit

## Discussion

To date there is no standardized SpO_2_ interval, universally recognized as excellent by the scientific community, to guarantee tissue oxygenation in the NICU setting. Multicenter, randomized and large-scale clinical trials have shown SpO_2_ below 90%, during intensive care stay, are associated with a higher mortality, and saturation values higher than 95% are associated with increased morbidity [11, 12, 22]. The present study allowed to draw a representative picture of how the management of oxygen monitoring is performed in 93 neonatal intensive care units in Italy and which factors independently favored/refrained the use of the recommended targets. The study revealed a wide variability in the utilized ranges for the surveillance of critically ill patients. Failure to comply with the upper and lower limits inevitably exposes the baby to either hypoxia or hyperoxia. Our study showed that in 64.4% of the centers the lower limit of SpO_2_ is set below 90%, highlighting a propensity to tolerate hypoxia; in contrast, in 24.4% of the centers the upper limit of SpO_2_ is set above 95% reflecting a permissive attitude towards hyperoxia. In newborns hyperoxia leads to persistent inflammation with impaired innate immune response and increased airway reactivity and susceptibility to respiratory virus infections in adulthood [23]. To limits the dangerous effects of hypoxia or hyperoxia a strict control of oxygen administration is mandatory.

A recent paper investigating the signal type of alarms in the NICU, reported that over 60% of alarms were related to oxygenation monitoring; thus, these represent the major burden of all alarms in newborns population [24]. In this survey, the setting of the maximum value is influenced by the presence or absence of both written operational procedures and staff training, with attention to hyperoxia conditions. NICUs that are particularly sensitive to staff training on the monitoring of SpO_2_, choose more frequently not to disable the alarms during care maneuvers, compared to the centers with opposite attitude. In this way the risk of missing clinically relevant alarms is reduced. So, it is clear how scientific updating and the active involvement of the trained staff in guiding daily clinical decisions play a decisive role in the quality of patient care. In this context, the so called “alarm fatigue” or alarm desensitization is also a key factor. In the NICU environment, the number of alarm signals may reach several hundred per day determining a huge alarm burden with the effect of staff desensitization, disabling of alarm signals and missing upper alarms [25]. The high rates of false or nonactionable alarms may also be involved. In most cases, nurses/neonatologists can adjust the SpO_2_ alarm limits and that is reasonable to individualize care to specific patients. However, the presence of local standard protocols may be desirable to avoid individual operator changes to alarm settings. The optimal SpO_2_ range for the newborn is also a dynamic value that can change (minimum, maximum or both) in relation to comorbidity, the inspired fraction of oxygen, the gestational age of the newborn and the corrected age. In 18.7% of the centers, saturation limits were never changed. This data challenge current recommendation to increase the saturation target to> 95% in those newborns who, at 32 weeks of correct gestational age, still need oxygen supplementation or to reconsider saturation targets in babies with pulmonary hypertension [26, 27]. All these aspects are fundamental not only to guarantee optimal neonatal care but also for later follow-up assessment. The use of specific local protocols and ad hoc personnel training were independently associated with the use of recommended targets limits, indicating the use of these tools as a good local guideline for better neonatal care. A recent European survey, performed only one year before the one presented in this paper, reported a wide variability in ranges similar to our data [21]. Consistently with their findings, the present survey also highlighted a lack of consensus regarding SpO_2_ target limits for preterm infants. However, they did not take into account the nurses/infants ratio and the “alarm fatigue” and it was not clear if there was a tendency to tolerate hypoxia or a permissive attitude towards hyperoxia, as suggested by the present findings. Moreover, we could not draw conclusions regarding the changes in the neonatologists’ view on SpO_2_ monitoring since we did not take into account in our survey when the written internal protocol was eventually introduced.

### Limitations

The present study has some limitations. First, the questionnaire gives an instant picture of only 25% of the Italian centers. Unfortunately, we were only allowed to send a single reminder to the invited responders. This means that the present study gives only a partial view on the actual situation of SpO_2_ targets in the Italian NICUs. Furthermore, the absence of more detailed background information on the specific SpO_2_ monitoring protocols used in each individual institution, and above all the lacking information of clinical outcome limits further conclusions. Also, the absence of a standard local protocol cannot be automatically equated with the absence of knowledge on current guidelines or lower quality of patient care. However, the presence of ad hoc protocols was identified as one of the independent factors associated with the use of recommended SpO_2_ targets.

## Conclusions

The study reveals that SpO_2_ monitoring, although available and performed in all units, still lacks specific local ad hoc protocols which need to be implemented for a correct surveillance of critically ill patients. The use of local protocols, and specific personnel training would possibly allow the wider use of recommended targets limits. These data provide an important overview on the current situation on Italian NICUs on SpO_2_ monitoring and management.

## Data Availability

The datasets generated and/or analysed during the current study are not publicly available due privacy reasons but are available from the corresponding author on reasonable request.

## Abbreviations

ROS: Reactive oxygen species
SpO_2_: Oxygen saturation
NICU: Neonatal intensive care unit
eCRF: electronic survey

## References

[1] Buonocore G, Perrone S. Tataranno ML. Oxygen toxicity: chemistry and biology of reactive oxygen species. Semin Fetal Neonatal Med 2010;15:186–190, 4, DOI: 10.1016/j.siny.2010.04.003. 10.1016/j.siny.2010.04.003 20494636

[2] Perrone S, Laschi E, Buonocore G (2019). Biomarkers of oxidative stress in the fetus and newborns. Free Radic Biol Med.

[3] Belvisi E, Bracciali C, Ognean ML, Tei M, Negro S, Carra F, Proietti F, Buonocore G, Perrone S, on behalf of the “Gruppo di Studio di Biochimica Clinica Neonatale della Società Italiana di Neonatologia” (2016). Enzyme activities in erythrocytes of term and preterm newborns. J Pediatr Biochem.

[4] Perrone S, Vezzosi P, Longini M, Marzocchi B, Paffetti P, Bellieni CV (2009). Biomarkers of oxidative stress in babies at high risk for retinopathy of prematurity. Front Biosci (Elite Ed).

[5] Perrone S, Tataranno ML, Stazzoni G, Del Vecchio A, Buonocore G (2012). Oxidative injury in neonatal erythrocytes. J Matern Fetal Neonatal Med.

[6] Perrone S, Santacroce A, Longini M, Proietti F, Bazzini F, Buonocore G (2018). The free radical diseases of prematurity: from cellular mechanisms to bedside. Oxidative Med Cell Longev.

[7] Bachman TE, Newth CJL, Iyer NP, Ross PA, Khemani RG (2019). Hypoxemic and hyperoxemic likelihood in pulse oximetry ranges: NICU observational study. Arch Dis Child Fetal Neonatal Ed.

[8] Bizzarro MJ (2018). Optimizing oxygen saturation targets in extremely preterm infants. JAMA..

[9] Cummings JJ, Polin RA (2019). Oxygen targeting in extremely low birth weight infants -more progress needed. J Pediatr.

[10] Clucas L, Doyle LW, Dawson J, Donath S, Davis PG (2007). Compliance with alarm limits for pulse oximetry in very preterm infants. Pediatrics..

[11] Stenson BJ, Tarnow-Mordi WO, Darlow BA, BOOST II United Kingdom Collaborative Group; BOOST II Australia Collaborative Group; BOOST II New Zealand Collaborative Group (2013). Oxygen saturation and outcomes in preterm infants. N Engl J Med.

[12] Schmidt B, Whyte RK, Asztalos EV, Moddemann D, Poets C, Rabi Y, Solimano A, Roberts RS, Canadian Oxygen Trial (COT) Group (2013). Effects of targeting higher vs lower arterial oxygen saturations on death or disability in extremely preterm infants: a randomized clinical trial. JAMA..

[13] Tataranno ML, Oei JL, Perrone S, Wright IM, Smyth JP, Lui K, Tarnow-Mordi WO, Longini M, Proietti F, Negro S, Saugstad OD, Buonocore G (2015). Resuscitating preterm infants with 100% oxygen is associated with higher oxidative stress than room air. Acta Paediatr.

[14] Sweet DG, Carnielli V, Greisen G, Hallman M, Ozek E, Plavka R, Saugstad OD, Simeoni U, Speer CP, Vento M, Visser GHA, Halliday HL (2017). European consensus guidelines on the Management of Respiratory Distress Syndrome - 2016 update. Neonatology..

[15] Manja V, Lakshminrusimha S, Cook DJ (2015). Oxygen saturation target range for extremely preterm infants: a systematic review and meta­analysis. JAMA Pediatr.

[16] Simpson KR, Lyndon A (2019). False alarms and Overmonitoring: major factors in alarm fatigue among labor nurses. J Nurs Care Qual.

[17] Ketko AK, Martin CM, Nemshak MA, Niedner M, Vartanian RJ (2015). Balancing the tension between Hyperoxia prevention and alarm fatigue in the NICU. Pediatrics..

[18] Bonafide CP, Localio AR, Holmes JH, Nadkarni VM, Stemler S, MacMurchy M, Zander M, Roberts KE, Lin R, Keren R (2017). Video analysis of factors associated with response time to physiologic monitor alarms in a Children's hospital. JAMA Pediatr.

[19] Hagadorn JI, Sink DW, Buus-Frank ME, Edwards EM, Morrow KA, Horbar JD, Ferrelli K, Soll RF (2017). Alarm safety and oxygen saturation targets in the Vermont Oxford network iNICQ 2015 collaborative. J Perinatol.

[20] Bachman TE, Iyer NP, Newth CJL, Ross PA, Khemani RG (2020). Thresholds for oximetry alarms and target range in the NICU: an observational assessment based on likely oxygen tension and maturity. BMC Pediatr.

[21] Huizing MJ, Villamor-Martínez E, Vento M, Villamor E (2017). Pulse oximeter saturation target limits for preterm infants: a survey among European neonatal intensive care units. Eur J Pediatr.

[22] Barlow BA, Vento M, Beltempo M, Lehtonen L, Håkansson S, Reichman B (2018). Variations in oxygen saturation targeting, and retinopathy of prematurity screening and treatment criteria in neonatal intensive care units: an international survey. Neonatology.

[23] Kumar VH, Lakshminrusimha S, Kishkurno S, Paturi BS, Gugino SF, Nielsen L (2016). Neonatal hyperoxia increases airway reactivity and inflammation in adult mice. Pediatr Pulmonol.

[24] Li T, Matsushima M, Timpson W, Young S, Miedema D, Gupta M, Heldt T (2018). Epidemiology of patient monitoring alarms in the neonatal intensive care unit. J Perinatol.

[25] Johnson KR, Hagadorn JI, Sink DW (2017). Alarm safety and alarm fatigue. Clinics Perinatol.

[26] Walsh-Sukys MC, Tyson JE, Wright LL, Bauer CR, Korones SB, Stevenson DK, Verter J, Stoll BJ, Lemons JA, Papile LA, Shankaran S, Donovan EF, Oh W, Ehrenkranz RA, Fanaroff AA (2000). Persistent pulmonary hypertension of the newborn in the era before nitric oxide: practice variation and outcomes. Pediatrics.

[27] Askie LM, Darlow BA, Davis PG, Finer N, Stenson B, Vento M (2017). Effects of targeting lower versus higher arterial oxygen saturations on death or disability in preterm infants. Cochrane Database Syst Rev.

